# Targeting LRPPRC to improve corneal wound healing via augmenting autophagy and suppressing TGF-β/Smad signaling post-alkali burn

**DOI:** 10.1186/s12967-026-08653-6

**Published:** 2026-07-24

**Authors:** Bolin Chen, Huizhuo Xu, Bowen Li, Yingjun Cai, Lihui Xie, Jing Zou

**Affiliations:** 1 Eye Center of Xiangya Hospital, Hunan Key Laboratory of Ophthalmology, Central South University, No.87, Xiangya Road, Kaifu District, Changsha, Hunan Province 410008 China; 2 National Clinical Research Center for Geriatric Disorders, Xiangya Hospital, Central South University, Changsha, 410008 Hunan China

**Keywords:** Cornea, LRPPRC, Autophagy, Fibrosis, ECM

## Abstract

**Background:**

Following corneal injury, the body initiates wound healing to maintain corneal function. Activation of the transforming growth factor beta (TGF-β) pathway promotes corneal stromal fibroblasts differentiation, leading to the excessive secretion of extracellular matrix (ECM) components, such as fibronectin and collagen, which contribute to stromal deposition, scar formation, and vision impairment. Although autophagy, a key regulatory pathway in corneal alkali burn repair, is critically modulated by the autophagy-inhibiting protein Leucine-Rich Pentatricopeptide Repeat Containing (LRPPRC), its specific role in this reparative process remains unclear.

**Method:**

A corneal alkali burn mouse model was established, and day 14 post-injury was selected as the primary time point for analysis (*n* = 6 per group). The expression and distribution of LRPPRC were assessed by Western blot and immunofluorescence (IF). To investigate the effects of LRPPRC downregulation on corneal injury, anterior chamber injection of LRPPRC-shRNA and ocular surface application of the LRPPRC-specific inhibitor Gossypol-Acetic Acid (GAA) eye drops were administered. Corneal opacity was assessed by slit-lamp photography, while fluorescein sodium staining evaluated the area of corneal damage. Anterior segment optical coherence tomography (OCT) and H&E staining measured corneal thickness and the degree of angle closure. CD31 staining assessed corneal neovascularization. The expression of the TGF-β pathway, ECM proteins, autophagy-related proteins, and inflammatory factors were evaluated by Western blot and IF.

**Result:**

Mice with corneal alkali burns exhibited characteristic pathological features, including severe corneal edema, neovascularization, and anterior chamber angle closure, and the expression level of LRPPRC exhibited a time-dependent increase. Following LRPPRC knockdown via shRNA or pharmacological inhibition with GAA, treated corneas demonstrated significant alleviation of edema and neovascularization, along with attenuated anterior chamber angle closure. Mechanistically, LRPPRC silencing suppressed TGF-β/Smad pathway, fibroblast activation and ECM deposition (e.g., fibronectin), while simultaneously enhancing autophagy-related protein expression, thereby facilitating ECM degradation during corneal repair. Notably, GAA treatment suppressed TGF-β-driven fibrotic responses in human corneal stromal cells (HCSCs), characterized by diminished expression of α-SMA, collagen, and fibronectin aggregates.

**Conclusions:**

LRPPRC inhibition demonstrated efficacy in alleviating pathological corneal fibrosis and neovascularization via suppression of fibroblast activation and ECM deposition post-corneal injury. These findings highlight LRPPRC as a tractable therapeutic target for corneal alkali burns, with GAA as a pharmacological agent offering a clinically translatable intervention.

**Supplementary Information:**

The online version contains supplementary material available at https://doi.org/10.1186/s12967-026-08653-6.

## Background

The cornea is a transparent, avascular membrane-like tissue located at the anterior surface of the eye. Its transparency is crucial for maintaining refractive function. When the cornea is damaged due to trauma or harmful stimuli, the body initiates a physiological response known as corneal wound healing to maintain its normal function [1]. This process can be complicated by various factors, including stromal opacification, surface irregularities and corneal perforation, which can significantly impair vision and potentially result in blindness [2, 3]. Among these pathological changes, excessive stromal fibrosis and abnormal ECM accumulation are the primary causes of permanent corneal opacity. Therefore, identifying the key regulatory molecules and mechanisms in corneal wound healing is essential to promote corneal repair and reduce vision loss following injury.

Corneal wound healing is a complex biological process involving the proliferation and migration of corneal epithelial cells, the activation and transformation of stromal cells, the deposition of extracellular matrix (ECM), the infiltration of inflammatory cells, and neovascularization [4]. Numerous molecules, including cytokines, transcription factors, and enzymes, are involved in corneal wound healing [5]. Transforming growth factor beta(TGF-β), a key profibrotic cytokine released by damaged corneal epithelial cells, has been identified as the potent inducer of myofibroblast transformation in corneal keratocytes [6]. Following epithelial injury, elevated levels of TGF-β penetrate the stroma and drive the activation of quiescent keratocytes into fibroblasts, which subsequently differentiate into myofibroblasts under sustained TGF-β signaling. These activated stromal cells represent the principal source of ECM synthesis during corneal wound healing [7]. The ECM, acting as a bridge for cell-cell communication, regulates physiological processes such as cell proliferation, differentiation, adhesion, spreading, and migration, thereby significantly contributing to corneal repair [8]. As a major component of the corneal tissue, the ECM provides mechanical support and connections between cells, imparting tensile strength and resilience to protect ocular contents. Furthermore, it plays a crucial role in maintaining corneal transparency and ensuring its refractive function. Following corneal injury, a large number of stromal cells are activated into corneal fibroblasts, which are the primary cellular participants in wound healing and serve as an important source of ECM synthesis during this process. However, excessive fibroblast activation and intense inflammatory response are induced, causing large amounts of ECM to accumulate and producing scar tissue [9].

Corneal fibrosis results not only from excessive ECM synthesis, but also from insufficient degradation of accumulated ECM components. Autophagy plays a critical regulatory role during the process of corneal healing by modulating inflammation and neovascularization. Previous studies have demonstrated that rapamycin, an autophagy agonist, reduces inflammatory cell infiltration following alkali burns, thereby facilitating corneal repair [10, 11]. Additionally, silencing S100 calcium-binding protein A4(S100A4) promotes corneal repair by enhancing autophagy in alkali-burned corneas [12]. Accumulating evidence suggests that autophagy is a critical mechanism for maintaining ECM homeostasis by facilitating the degradation of excessive or abnormal matrix components. Autophagy is associated with cellular ECM degradation, in which Microtubule-associated protein 1 light chain 3(MAP1S) plays a role in ECM degradation in liver fibrosis [13]. Leucine-Rich Pentatricopeptide Repeat Containing (LRPPRC), a mitochondrial protein that binds to MAP1S, is closely related to the initiation of autophagy [14–16]. However, whether LRPPRC participates in corneal wound healing and stromal fibrosis by regulating autophagy-dependent ECM turnover and profibrotic signaling pathways remains unknown. Therefore, we investigated the role of LRPPRC in ECM deposition after corneal injury. Initially, we examined the expression changes of LRPPRC, autophagy-related proteins, and ECM components in alkali-burned corneas. Subsequently, we employed anterior chamber injection of LRPPRC-shRNA and topical application of Gossypol-Acetic Acid (GAA), a specific LRPPRC inhibitor, to assess its effect on corneal autophagy levels, ECM deposition and corneal injury repair. Furthermore, we explored the underlying mechanisms by which LRPPRC regulates both TGF-β/Smad signaling and autophagy-mediated ECM degradation in human corneal stromal cells (HCSCs). Based on these studies, we aim to determine whether LRPPRC serves as a key regulator in corneal injury repair and whether it could be a potential therapeutic target.

## Materials and methods

### Cell culture

Human corneal stromal cells (HCSCs) were purchased from Abiowell Company (AW-YCH154, Changsha, China) and cultured in DMEM/F-12 (Gibco, USA). All the media were supplemented with 2% fetal bovine serum (FBS) and 1% penicillin/streptomycin unless specifically stated. Cells were maintained at 37℃ and 5% CO_2_ in a humidified incubator.

To induce a fibrotic phenotype, HCSCs were cultured in medium containing 0.1% FBS and stimulated with 10µM TGF-β (HY-P70543. MCE, USA) for 7 days. For pharmacological intervention, cells were treated with Gossypol Acetate (GAA, s2303, Selleck, USA) at the indicated concentration. Chloroquine (CQ, 10µM, HY-17589 A, MCE, USA) was applied as a lysosomal inhibitor to block autophagic flux, either alone or in combination with GAA, to assess whether the effects of GAA were dependent on autophagy. Cells treated with vehicle served as controls.

### Animals and alkali burn mouse model

In this study, male C57BL/6 mice aged 6–8 weeks were used, obtained from Hunan SJA Laboratory Animal Co., Ltd. (Changsha, China). The mice were fed under specific pathogen-free (SPF) conditions at the Experimental Animal Center of Xiangya Hospital, Central South University. All procedures involving animals were conducted in strict accordance with the ARVO Statement for the Use of Animals in Ophthalmic and Vision Research. The animal experiments were approved by the Animal Ethics Committee of Xiangya Hospital, Central South University (Approval No.202411184). Mice were anaesthetized via intraperitoneal injection of 1% sodium pentobarbital, followed by topical anaesthesia of the eyes with 1% proparacaine. A 2 mm diameter circular filter paper, pre-soaked in 1 M NaOH, was blotted dry for 5 s and then applied to the central cornea for 30 s. The eye was immediately rinsed with 0.9% saline for 60 s. Mice were randomly assigned to different experimental groups depending on the study design. For the LRPPRC knockdown experiments, mice were divided into four groups (*n* = 6 per group): Normal, Control, NC-shRNA, and LRPPRC-shRNA. For the LRPPRC inhibitor intervention experiments, mice were divided into four groups (*n* = 6 per group): Saline, 5µM GAA, 10µM GAA, and 20µM GAA. Adenoviral injection into the anterior chamber was performed 7 days before corneal alkali burn injury. Corneal tissues were collected at the indicated time points for subsequent analyses.

### Anterior chamber injection

Mice were anaesthetized with 1% sodium pentobarbital, followed by topical anaesthesia of the ocular surface with proparacaine eye drops. Under a stereomicroscope, a 33G microsyringe (Hamilton, Germany) was used to create a fistula by inserting the needle parallel to the corneal limbus. A cotton swab was used to absorb the aqueous humor, after which the needle was reinserted to inject 1 µL of adenovirus into the anterior chamber [17]. Construction of AAV-shRNA targeting LRPPRC Short hairpin RNA (shRNA) targeting mouse LRPPRC was designed based on the siRNA target sequence 5’-GCGTGGAACTTGAAACAAGAA-3’ (GC content: 36.84%). The shRNA oligonucleotides were cloned into the GV478 vector, in which shRNA expression was driven by the U6 promoter. A scrambled shRNA sequence (5’-CGCTGAGTACTTCGAAATGTC-3’) was used as a negative control. All constructs were sequence-verified before virus packaging. Adenoviral vectors encoding LRPPRC-shRNA and negative control (NC-shRNA) were constructed and purified by Baitaike Bio (Changsha, China). The viral titre was 1.01 × 10^12^ plaque-forming units (PFU)/ml, as provided by the company. Adenovirus encoding LRPPRC-shRNA or NC-shRNA (1.57 × 10^11^ PFU/ml) alone was injected into the anterior chamber of the mice 7 days before alkali burn injury. Following the injection, erythromycin ointment was applied to the ocular surface to prevent infection. The mice were allowed to recover in a warmed environment and were subsequently fed under normal room oxygen conditions.

### Clinical assessment

The ocular surface was observed and assessed under a microscope at predetermined time points, with images captured using an attached camera for corneal evaluation. Two independent observers scored corneal opacity and neovascularization size, and the final score was determined by averaging their scores. The corneal injury was assessed using established criteria:

1. Corneal opacity was graded with a score of 0 to 4, where 0 = clear cornea; 1 = slight haze, pupil visible; 2 = moderate opacity, but pupil detectable; 3 = severe opacity, pupil hardly detectable; and 4 = complete opacity, pupil undetectable.
2. Corneal neovascularization (CNV) was graded with a score of 0 to 3, where 0 = no CNV; 1 = CNV within the corneal limbus; 2 = CNV over the corneal limbus to the corneal centre; and 3 = CNV within the corneal centre.
3. Neovessel size was graded with a score of 0 to 3, where 0 = no detectable neovessels; 1 = neovessels detectable under a microscope; 2 = neovessels easily seen under a microscope; and 3 = neovessels easily seen without a microscope.

If cornea perforation occurred, the total clinical assessment score was rated as 10. CNV was calculated as follows: S = C/12 × 3.1416 ×[R^2^ -(R-L)^2^] [18]. In this formula, S represented the CNV area, C represented the clock hour points covered by the CNV, R represented the radius of the cornea, and L represented the longest length of the neovessel.

### Optical Coherence Tomography (OCT)

After anaesthetizing the mice, a viscoelastic agent (Alcon, USA) was applied to the corneas to prevent dehydration. The mice were then positioned on a holder, and the lens of Optical Coherence Tomography (OCT, TowardPi, China) was aligned with the eye and slowly advanced, carefully adjusting the angle to centre the eye within the lens. After adjusting the gamma values in the accompanying software, anterior segment OCT and anterior chamber angle OCT images were acquired. The separation distance between the corneal endothelial border and the iris root was measured using ImageJ, and changes in the anterior chamber angle were graded according to the Shaffer classification.

### Western blot

Following a cervical dislocation, the mouse eyes were enucleated, and the corneas were carefully dissected under a microscope and placed into centrifuge tubes. RIPA lysis buffer was added to prepare a homogenate, which was lysed on ice for 30 min. The lysate was centrifuged, and the supernatant was collected. Protein samples were separated using SDS-PAGE and subsequently transferred onto a PVDF membrane. The membrane was blocked with 5% non-fat milk at room temperature. After overnight incubation with the anti-LRPPRC (21175-1-AP, Proteintech, China), anti-P62(18420-1-AP, Proteintech, China), anti-LC3 (AWA10125, Abiowell, China), anti-Bcl2(26593-1-AP, Proteintech, China), anti-Beclin 1(11306-1-AP, Proteintech, China), anti-IL-1β (16806-1-AP, Proteintech, China), anti-IL-6 (A0286, Abclonal, China), anti-TGF-β (21898-1-AP, Proteintech, China), anti-phospho-Smad2/3 (#8828, CST, USA), anti-Smad2/3 (R382472, Zenbio, China), anti-Fibronectin (15613-1-AP, Proteintech, China), anti-α-SMA (ab5694, Abcam, USA), anti-Integrin β1 (12594-1-AP, Proteintech, China), anti-Collagen I (14695-1-AP, Proteintech, China), anti-Collagen III(22734-1-AP, Proteintech, China), anti-VEGFA (19003-1-AP, Proteintech, China), anti-β-actin (66009-1-Ig, Proteintech, China) and anti-GAPDH (AP0066, Bioworld, USA) primary antibodies at 4 °C, respectively. The membrane was incubated with the HRP-conjugated Goat Anti-Rabbit IgG(H + L) (SA00001-2, Proteintech, China) and HRP-conjugated Goat Anti-Mouse IgG(H + L) (SA00001-1, Proteintech, China) secondary antibody at room temperature for 1 h. Finally, protein bands were visualized using the ChemiDoc system (BIO-RAD MP500, USA).

### Hematoxylin and eosin (H&E) staining

Eyeballs were fixed in 4% PFA, and serial sagittal Sect.  (5 μm thick) were made through the optic nerve head. The tissue sections were then stained with hematoxylin and eosin (H&E). Retinal morphology images were captured using a light microscope. Corneal thickness was measured at the center of the cornea with measurements facilitated by Case Viewer 2.4 software.

### Immunofluorescence staining

Eyeballs were harvested and immediately fixed in 4% paraformaldehyde at 4 °C. After fixation, tissues were immersed in 30% sucrose solution at 4 °C until they sank. The tissues were then embedded in an optimal cutting temperature (OCT) compound, frozen, and sectioned into 10 μm-thick slices using a cryostat. For staining, sections were first permeabilized with 0.3% Triton X-100, followed by blocking in 5% normal goat serum at room temperature. The sections were then incubated overnight at 4 °C with anti-LRPPRC (21175-1-AP, Proteintech, China), anti-P62(18420-1-AP, Proteintech, China), anti-LC3 (AWA10125, Abiowell, China), anti-IL-1β (16806-1-AP, Proteintech, China), anti-IL-6 (A0286, Abclonal, China), anti-TGF-β (21898-1-AP, Proteintech, China), anti-Vimentin(ab92547, Abcam, USA), anti-α-SMA (ab5694, Abcam, USA), anti-LAMP2 (10397-1-AP, Proteintech, China) and anti-Collagen I (14695-1-AP, Proteintech, China) primary antibodies. Sections were incubated with Alexa Fluor 488 goat anti-rabbit IgG (H + L) (A11008; Invitrogen) secondary antibodies for 1 h at room temperature in the dark. The nuclei were counterstained with 4′,6-diamidino-2-phenylindole (DAPI) for 10 min and coverslipped. Images were acquired using a fluorescence microscope (Zeiss) equipped with appropriate filters. Image analysis and quantification were performed using ImageJ software.

### Corneal neovascularization staining

After euthanizing the mice by cervical dislocation, the eyeballs were enucleated and fixed in 4% PFA at 4 °C overnight. The corneas were carefully dissected under a microscope and incised at the 3, 6, 9, and 12 o’clock positions using ophthalmic scissors. The corneas were then incubated in a solution of 3% Triton X-100 and 5% bovine serum albumin (BSA) overnight at 4 °C. Subsequently, the corneas were incubated with rat anti-mouse CD31 antibody (553370, BD Pharmingen, USA) at 4 °C for 48 h. Afterwards, the corneas were incubated with goat anti-rat 594-conjugated secondary antibody (A11007, ThermoFisher) at 4 °C for 24 h. Following antibody incubation, the corneas were then flat-mounted on glass slides and covered with coverslips. Images were captured under a fluorescence microscope. Fluorescence images of corneal flat mounts were analyzed using ImageJ software (National Institutes of Health, Bethesda, MD) [19].

### Statistical analysis

All statistical analyses were performed using GraphPad Prism (version 9.2; GraphPad Software). Data are presented as mean ± standard deviation (SD). Comparisons between two groups were made using the two-tailed Student’s t-test, while comparisons among multiple groups were performed using one-way or two-way analysis of variance (ANOVA). A p-value of < 0.05 was considered statistically significant.

## Results

### The repair process of corneal alkali burn in mice

**Fig. 1:**
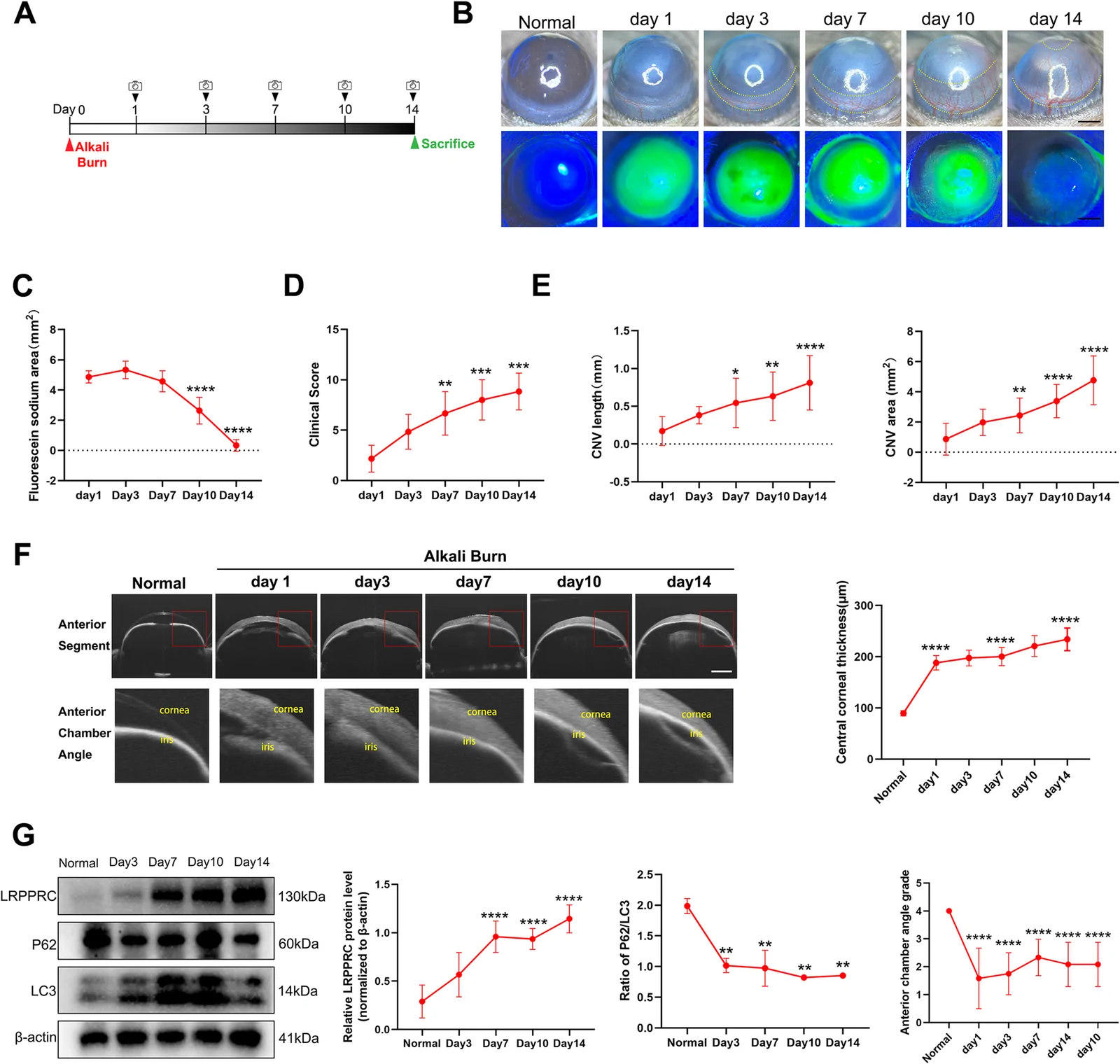
The corneal repair process in alkali burn mice. (**A**) Schematic diagram of modeling, and a photograph of a time point in mice. (**B**) Sodium fluorescein staining and anterior view of the anterior ocular segment; scale bars, 1 mm; the yellow dashed line indicates the area of CNV. (**C**) Measured statistics of fluorescein sodium staining range (*n* = 6). (**D**) Clinical assessment scores were measured in mice (*n* = 6). (**E**) Measured statistics of CNV length and CNV area (*n* = 6). (**F**) Representative image of anterior segment OCT of corneal alkali burn mice; scale bars, 400 μm. Quantification of central corneal thickness is shown (*n* = 6). (**G**) Western blot and analysis of LRPPRC and autophagy-related protein levels in cornea. CNV, corneal neovascularization. The presented data depict the average and standard deviation from a minimum of three repetitions, with differences assessed using standard one-way ANOVA. *p < 0.05, **p < 0.01, ***p < 0.001

Figure 1a illustrates a schematic overview of our in vivo modeling workflow and the subsequent assessment process. Eight-week-old male mice after alkali burn injury, demonstrate typical neovascularization (CNV) and a gradually expanding fluorescein sodium area starting from day 1 (Fig. 1B-C). In addition, the clinical corneal assessment score, based on CNV size, CNV area and corneal opacity increased gradually from day 3 post-alkali burn and onwards (Fig. 1D). To further provide an objective and quantitative evaluation of angiogenesis, CNV length and area were measured separately and are shown in Fig. 1E. Apart from causing severe damage to the cornea, alkali burn can result in the closure of the anterior chamber angle. To assess temporal changes in the architecture of the anterior segment of the eye post-alkali burn, anterior segment Optical Coherence Tomography (OCT) was employed to visualize corneal and anterior chamber angle structures. Central corneal thickness was increased significantly after alkali burn and the anterior chamber angle gradually closed. The iris root and the corneal endothelium border in complete adhesion, leaving no void space between them. The Shaffer grading system was used to analyze the degree of anterior chamber angle closure at different time points after alkali burn (Fig. 1F). Western blot analysis revealed a continuous upregulation of LRPPRC with prolonged repair duration, suggesting that LRPPRC plays an important role during the late phase of the repair process. Concurrently, the P62/LC3 ratio—recognized as the gold standard for evaluating autophagy activity—gradually declined over time, indicating enhanced autophagic activity. However, both P62 and LC3 levels decreased after day 10, implying an alteration in autophagic activity (Fig. 1G). These findings suggest a potential association between elevated LRPPRC and changes in autophagy during the late repair process.

### Downregulation of LRPPRC promotes corneal repair

**Fig. 2:**
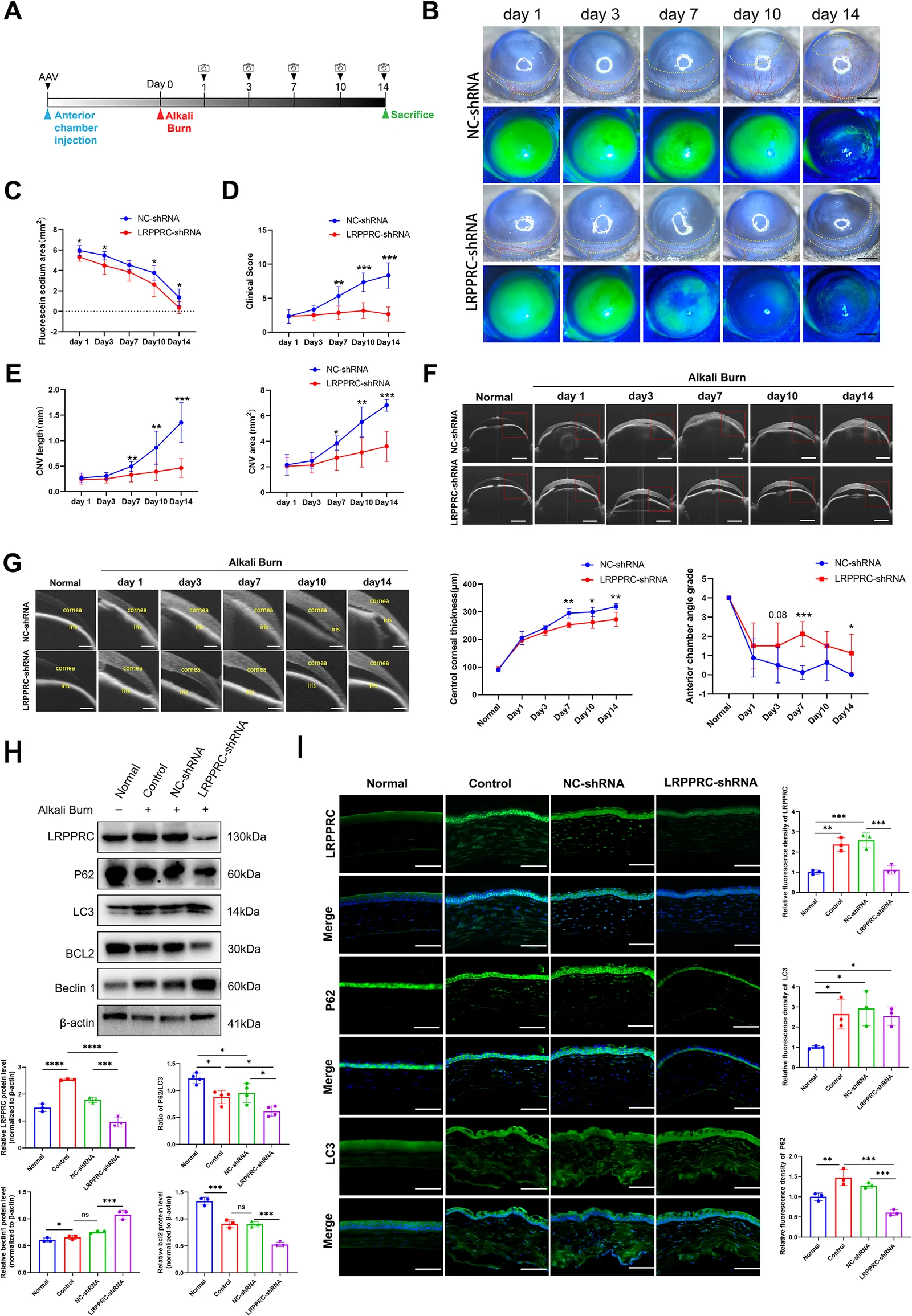
Inhibition of LRPPRC promotes corneal injury repair. (**A**) Schematic representation of anterior chamber injection, modeling and timepoints for photography in mice. (**B**) Sodium fluorescein staining and anterior view of the anterior ocular segment; scale bars, 1 mm; the yellow dashed line indicates the area of CNV. (**C**) Measured statistics of fluorescein sodium staining range (*n* = 6). (**D**) Clinical assessment scores for corneal injury (*n* = 6). (**E**) Measured statistics of CNV length and area (*n* = 6). (**F**) Representative anterior segment OCT images; scale bars, 400 μm. Central corneal thickness quantification is shown (*n* = 6). (**G**) Representative chamber angle OCT images; scale bars, 100 μm. Chamber angle grade quantification is shown (*n* = 6). (**H**) Western blot and analysis of LRPPRC and autophagy-related protein in the cornea (*n* = 3). (**I**) Immunostaining of LRPPRC, P62, and LC3 expressing in the corneal of four groups; scale bars, 10 μm. Fluorescence intensities were quantified using Image J software (*n* = 3). Normal: healthy; Control: corneal alkali burn; NC-shRNA: anterior chamber injection negative control; LRPPRC-shRNA: anterior chamber injection LRPPRC-shRNA; CNV, corneal neovascularization. The presented data depict the average and standard deviation from a minimum of three repetitions, with differences assessed using standard one-way ANOVA. *p < 0.05, **p < 0.01, ***p < 0.001

Prior to the formal injury experiments, a pilot time-course study was performed to evaluate the knockdown efficiency of LRPPRC following anterior chamber injection of LRPPRC-shRNA. Corneal tissues were collected at day 3, day 5, day 7, day 10, and day 14 after injection, and LRPPRC protein levels were assessed by Western blot (Supplementary Figure 1A). These results demonstrated that LRPPRC expression was markedly reduced from day 5 onward, with stable suppression maintained through day 7. Based on these findings, day 7 post-injection was selected as the optimal time point for alkali burn induction. Figure 2A illustrates a schematic diagram of anterior chamber injection, alkali burn and photograph workflow. Fourteen days after alkali burn injury, mice in the LRPPRC-shRNA group exhibited a smaller area of sodium fluorescein staining, reduced corneal neovascularization length and area, and lower clinical corneal assessment score compared to the NC-shRNA group (Fig. 2B-E). OCT analysis revealed a decrease in corneal thickness, reduced anterior chamber angle closure, and a lower Shaffer grading in the LRPPRC-shRNA group compared to the NC-shRNA group (Fig. 2F-G). Western blot analysis demonstrated a relative decrease in the P62/LC3 ratio following LRPPRC knockdown, indicating increased autophagic activity (Fig. 2H). Consistently, the expression of the autophagy initiation protein Beclin1 was markedly increased, whereas the anti-autophagic protein Bcl-2 was significantly decreased after LRPPRC-shRNA administration, further supporting autophagy activation in the cornea. Immunofluorescence also confirmed that decreased LRPPRC expression in the entire corneal layer was associated with increased LC3 and decreased P62 levels (Fig. 2I). These findings suggest that the downregulation of LRPPRC promotes autophagy.

### Inhibition of LRPPRC reduces inflammation and fibroblast activation via suppression of TGF-β expression

**Fig. 3:**
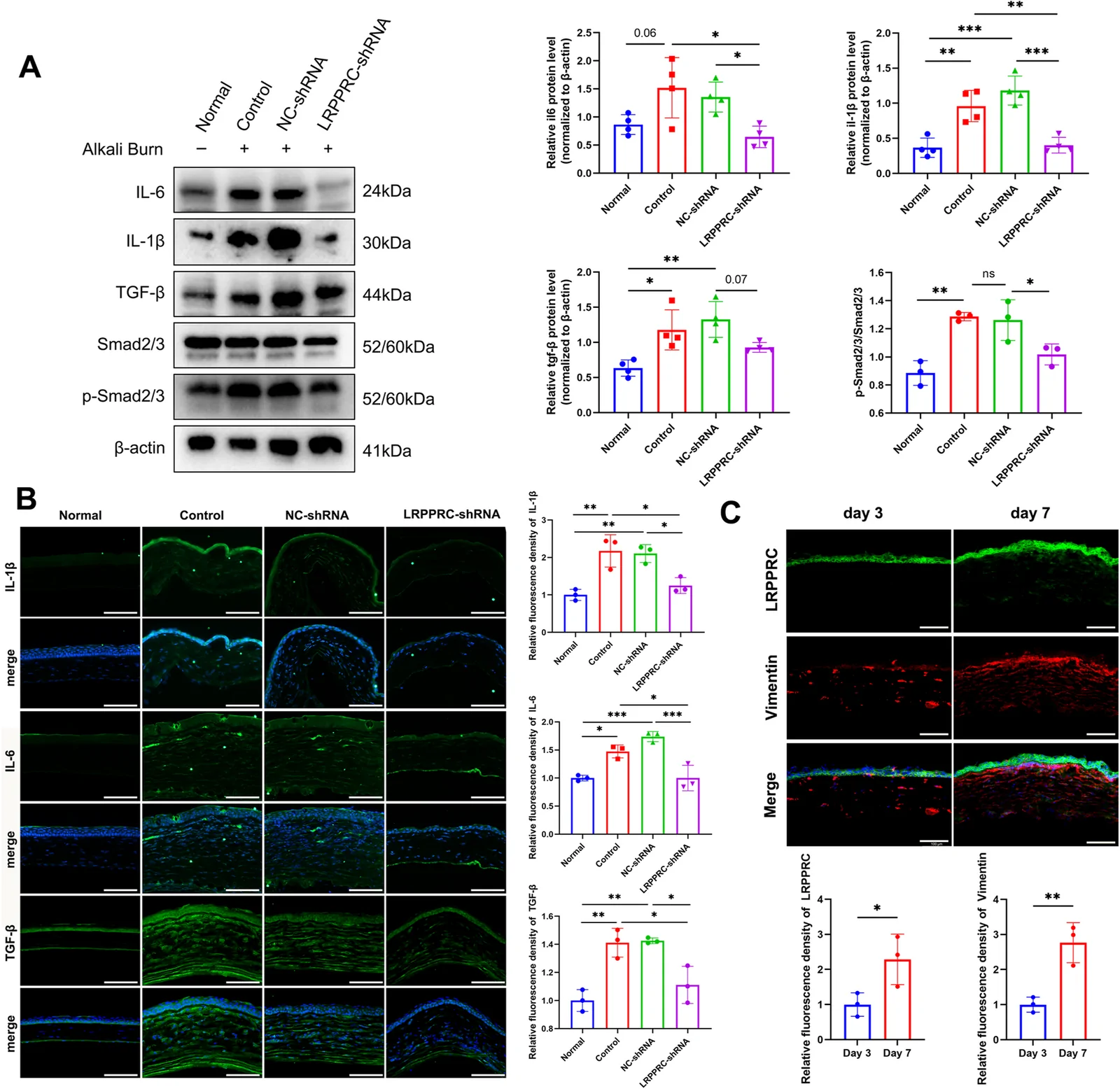
Inhibition of LRPPRC reduces inflammation and TGF-β expression (**A**) Western blot and analysis of inflammation factors and TGF-β signaling pathway protein levels in the cornea (*n* = 4). (**B**) Immunostaining of inflammation factors expressed in the corneal of four groups; scale bars, 100 μm. Fluorescence intensities were quantified using Image J software (*n* = 3). (**C**) Immunostaining of LRPPRC and fibroblast marker Vimentin expressed in the corneal; scale bars, 100 μm. Fluorescence intensities were quantified using Image J software (*n* = 3). Normal: healthy; Control: corneal alkali burn; NC-shRNA: anterior chamber injection negative control; LRPPRC-shRNA: anterior chamber injection LRPPRC-shRNA. The presented data depict the average and standard deviation from a minimum of three repetitions, with differences assessed using standard one-way ANOVA. *p < 0.05, **p < 0.01, ***p < 0.001

To investigate the mechanism by which LRPPRC promotes corneal repair, we examined the inflammation factor and the classical TGF-β signaling, which is a key regulator of corneal stromal cells are activated and converted into myofibroblasts. Western blot analysis demonstrated that the expression of inflammatory factors, TGF-β, and the phosphorylation levels of Smad2/3 were markedly increased in the Control and NC-shRNA groups, whereas these elevations were significantly suppressed in the LRPPRC-shRNA group (Fig. 3A). Consistently, immunofluorescence staining revealed pronounced upregulation of inflammatory factors and TGF-β in the corneal stroma of the Control and NC-shRNA groups, which was substantially attenuated following LRPPRC knockdown (Fig. 3B). Given that activation of the TGF-β/Smad pathway promotes fibroblast activation during corneal fibrosis, we further performed immunofluorescence staining for the fibroblast marker vimentin. Increased vimentin expression was observed in the corneal stroma on days 3 and 7 after injury, indicating fibroblast activation during corneal repair. Notably, this increase was associated with elevated LRPPRC expression, suggesting that LRPPRC may contribute to corneal fibrosis by promoting fibroblast activation (Fig. 3C).

### Regulation of LRPPRC suppresses fibroblast overproliferation and reduces extracellular matrix deposition

To explore the effects of LRPPRC on fibroblast activation and ECM deposition, we performed HE staining on corneal samples from four groups of mice. The Control group exhibited significant corneal edema, with marked increases in both epithelial and stromal thickness compared to the healthy group, as confirmed by quantitative analysis of central corneal thickness. Similarly, corneal opacity and hemorrhage were observed in both the Control and NC-shRNA groups, rendering the background pattern visually indistinct. In contrast, the LRPPRC-shRNA group showed partial improvement in corneal edema, with a statistically significant reduction in central corneal thickness. Although inflammatory cell infiltration and corneal neovascularization (CNV) persisted, the corneas displayed moderate opacity, and the background pattern was more discernible (Fig. 3A-B). Subsequent analysis of ECM protein levels in the corneas revealed that fibronectin, α-SMA, integrin β1, collagen I and collagen III were significantly elevated in the Control and NC-shRNA groups compared to the normal group, while these protein levels were reduced in the LRPPRC-shRNA group (Fig. 3C). These findings suggest that LRPPRC modulation can inhibit fibroblast activation and reduce corneal ECM deposition, thereby alleviating delayed corneal repair and preventing vision impairment due to corneal opacity.

**Fig. 4:**
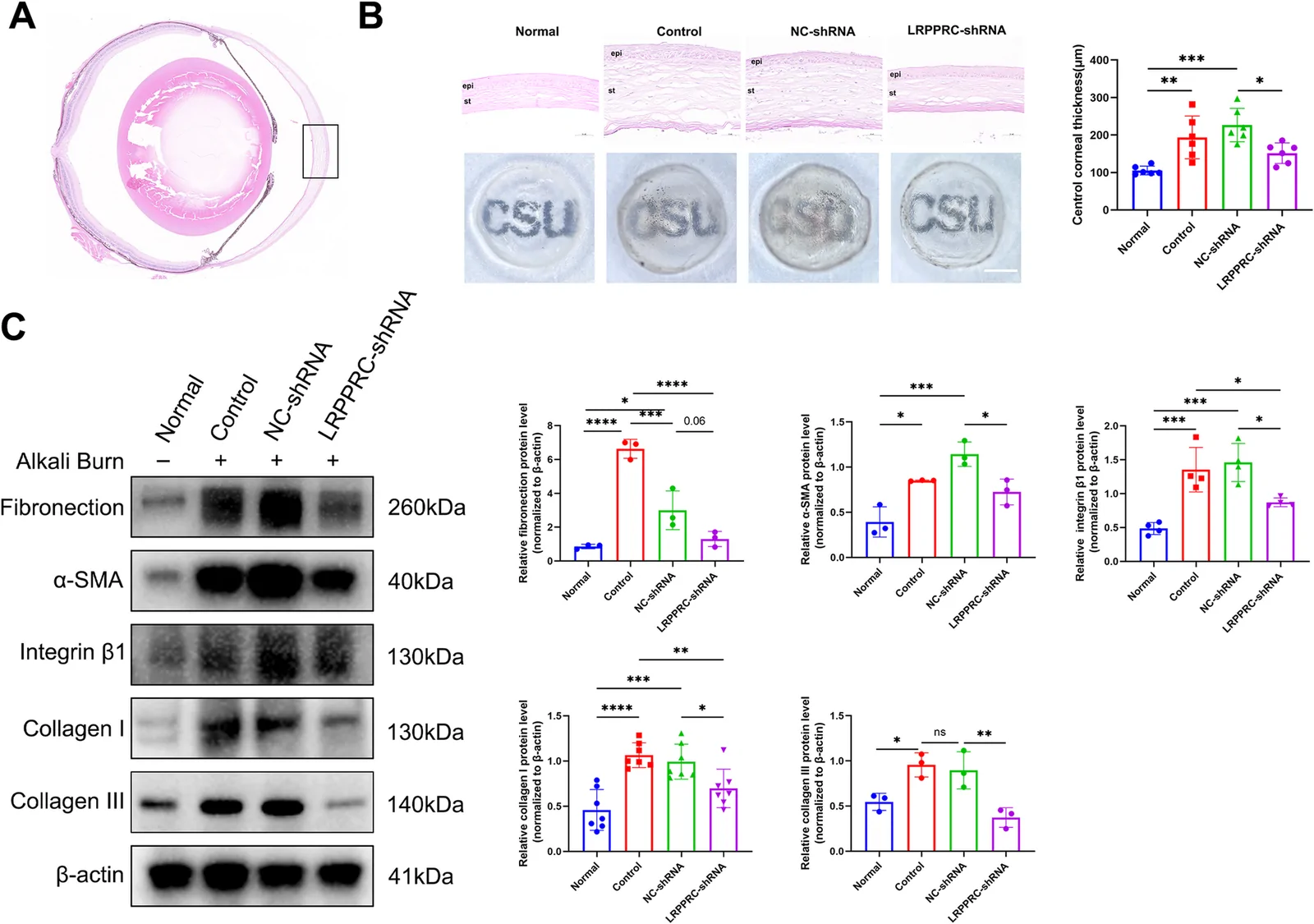
Inhibition of LRPPRC reduces ECM proteins and fibrosis. (**A**) HE staining schematic diagram of mouse eyeball section (**B**) Representative corneal H&E staining images for the four groups. Quantitative analysis of central corneal thickness. Scale bar, 50 μm (*n* = 6). Cornea images after sacrificing and dissecting on day 14. Scale bars, 1 mm. (**C**) Western blot and analysis of ECM-related protein levels in cornea. Normal: healthy; Control: corneal alkali burn; NC-shRNA: anterior chamber injection negative control; LRPPRC-shRNA: anterior chamber injection LRPPRC-shRNA. The presented data depict the average and standard deviation from a minimum of three repetitions, with differences assessed using standard one-way ANOVA. *p < 0.05, **p < 0.01, ***p < 0.001

### Reduced LRPPRC expression inhibits corneal neovascularization

Neovascularization is a critical factor influencing corneal transparency and repair. Compared to normal mice, those subjected to alkali burn exhibited a significant increase in neovascularization. Western blot analysis indicated that VEGFA levels were relatively elevated following alkali burn but were reduced in the LRPPRC-shRNA group (Fig. 4A). Similarly, CD31 staining revealed a marked increase in neovascularization in the NC-shRNA group, while the LRPPRC-shRNA group showed relatively fewer new blood vessels (Fig. 4B). These findings, together with previous examination scores, suggest that downregulation of LRPPRC can inhibit neovascularization during the corneal repair process following alkali burn.

**Fig. 5:**
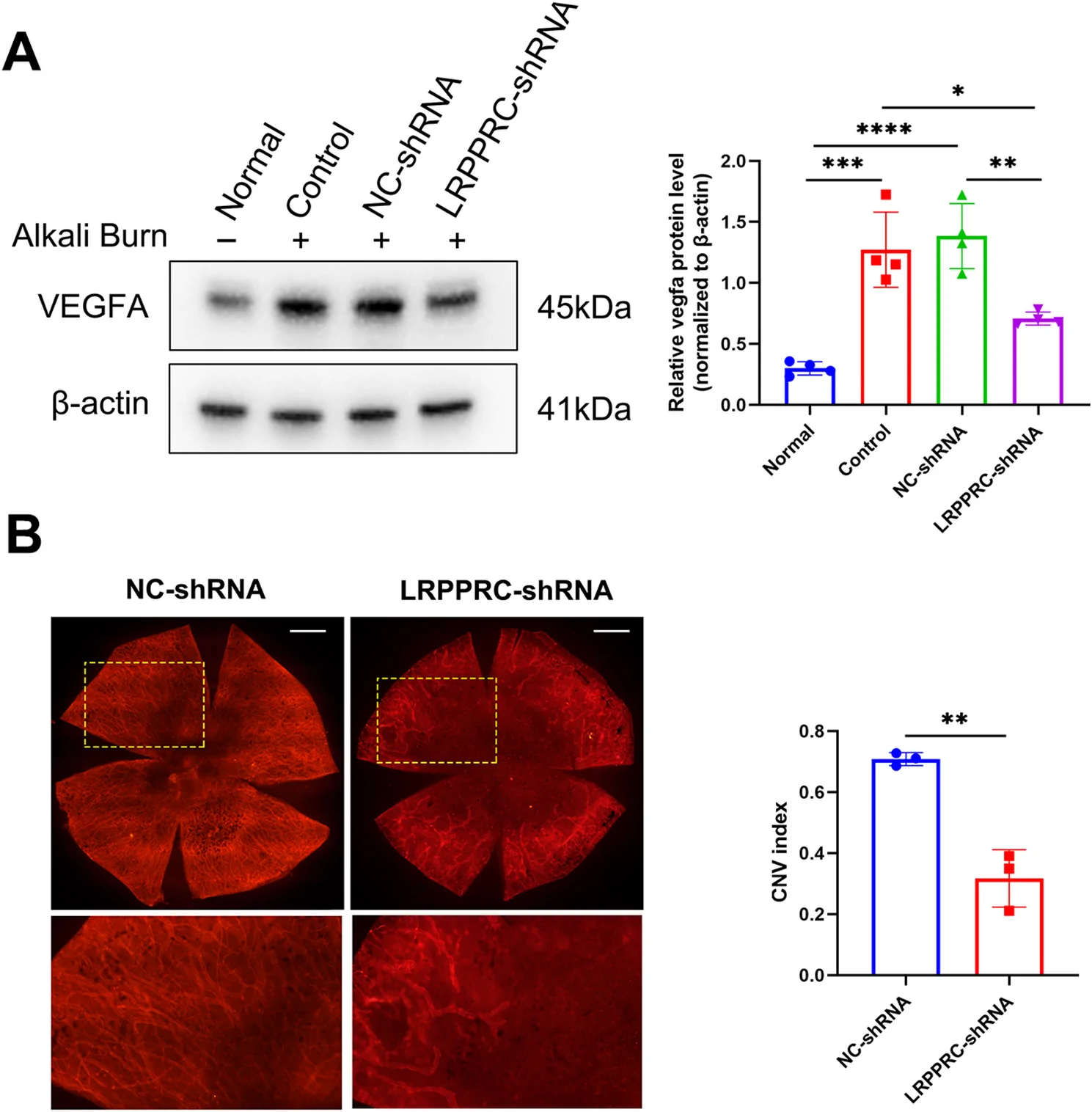
Inhibition of LRPPRC reduces corneal neovascularization. (**A**) Western blot and analysis of VEGFA levels in cornea(*n* = 4). (**B**) Corneal whole-mount CD31 staining and quantification showing that the NC-shRNA group exhibited notably increased neovascularization compared with the LRPPRC-shRNA group, scale bar: 500 μm, (*n* = 3). Normal: healthy; Control: corneal alkali burn; NC-shRNA: anterior chamber injection negative control; LRPPRC-shRNA: anterior chamber injection LRPPRC-shRNA. The presented data depict the average and standard deviation from a minimum of three repetitions, with differences assessed using standard one-way ANOVA. *p < 0.05, **p < 0.01, ***p < 0.001

### GAA-mediated LRPPRC inhibition accelerates corneal recovery

GAA is a specific inhibitor of LRPPRC, administered three times daily in mice. Slit-lamp imaging and fluorescein sodium staining demonstrated a dose-dependent effect of GAA on corneal repair compared to the saline group (Fig. 5A). HE staining revealed that treatment with 20 µM GAA significantly reduced corneal edema and corneal thickness in mice compared to the saline group (Fig. 5B). Quantitative analysis of sodium fluorescein staining further indicated that GAA treatment accelerates the speed of corneal repair, accompanied by lower clinical scores, as well as reduced area and length of CNV (Fig. 5C-E). Consequently, we selected the 20 µM GAA treatment for further evaluation using OCT, which showed that GAA significantly improved corneal thickness and anterior chamber angle closure compared to the saline group (Fig. 5F-G). CD31 staining and the degree of corneal opacity also suggested that GAA mitigated neovascularization and provided clearer background images in the alkali burn model (Fig. 5H). Western blot indicated that GAA treatment suppressed LRPPRC expression, accompanied by a marked reduction in inflammatory factors, attenuation of the TGF-β signaling pathway, and decreased expression of ECM components, while autophagy-related proteins were significantly upregulated (Fig. 5I), with quantitative data presented in Supplementary Figure 1B. Furthermore, immunofluorescence staining of corneal revealed that GAA treatment significantly reduced the expression of the pro-inflammatory cytokines IL-1β and IL-6 in the injured corneas, further confirming its anti-inflammatory effects (Supplementary Figure 1C). These findings suggest that GAA, as a specific inhibitor of LRPPRC, may facilitate corneal injury repair and hold potential for clinical application.

**Fig. 6:**
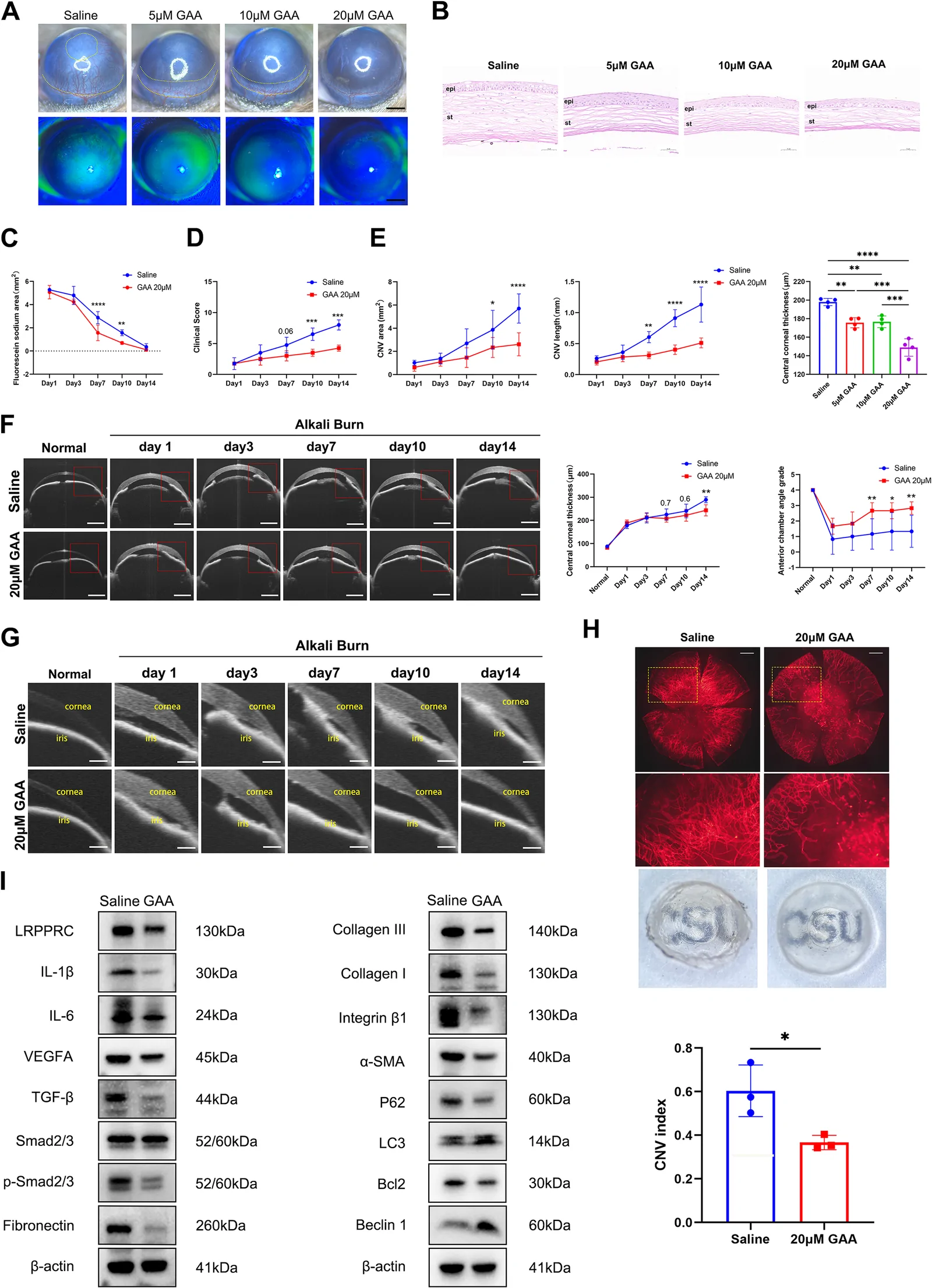
GAA accelerate corneal recovery. (**A**) Sodium fluorescein staining and anterior view of the anterior ocular segment in different concentrations of GAA; scale bars, 1 mm; the yellow dashed line indicates the area of CNV. (**B**) Representative corneal H&E staining images for the four groups and quantitative analysis of central corneal thickness. Scale bar, 50 μm. (*n* = 4). (**C**) Measured statistics of fluorescein sodium staining range (*n* = 4). (**D**) Clinical assessment scores were measured in mice (*n* = 4). (**E**) Measured statistics of CNV area and CNV length (*n* = 4). (**F**) Representative image of anterior segment OCT of saline and 20µM GAA, scale bars, 400 μm. Quantification of central corneal thickness is shown (*n* = 6). (**G**) Representative chamber angle OCT images; scale bars, 100 mm. Chamber angle grade quantification is shown (*n* = 6). (**H**) Corneal whole-mount CD31 staining and quantification showing that the Saline group exhibited notably increased neovascularization compared with the 20µM GAA group, scale bar: 500 μm, (*n* = 3). Cornea images after sacrificing and dissecting on day 14. Scale bars, 1 mm. (**I**) Western blot analysis of corneal tissues showing the effects of GAA on LRPPRC expression, inflammatory factors, TGF-β signaling, ECM components, and autophagy-related proteins (*n* = 3). The presented data depict the average and standard deviation from a minimum of three repetitions, with differences assessed using standard one-way ANOVA. *p < 0.05, **p < 0.01, ***p < 0.001

### GAA inhibits TGF-β/Smad–driven fibrosis and activates autophagy in human corneal stromal cells

We found that GAA treatment significantly downregulated LRPPRC expression in human corneal stromal cells (HCSCs) in a concentration-dependent manner (Fig. 6A). When HCSCs were cultured in a low-serum medium containing 10 µM TGF-β for seven days, phosphorylation of Smad2/3 was markedly increased, while total Smad2/3 levels remained unchanged, indicating activation of the TGF-β/Smad signaling pathway. Subsequently, significant increase in the expression of fibrosis-related proteins, including α-SMA, fibronectin, collagen I and collagen III, was significantly upregulated. Cellular immunofluorescence analysis further revealed that, following fibrosis induction, HCSCs exhibited increased cell volume, prominent α-SMA stress fiber formation, and enhanced accumulation of fibronectin and collagen I, resulting in a pronounced fibrotic phenotype. However, treatment with 2µM GAA markedly reversed these effects, leading to reduced expression of fibrosis-related proteins and attenuation of fibrotic cellular morphology, suggesting that GAA effectively inhibits TGF-β/Smad pathway activation and HCSC fibrosis (Fig. 6B-C).

To determine whether the effects of GAA were mediated through autophagy, chloroquine (CQ), an autophagy inhibitor that blocks lysosomal acidification and impairs autophagic flux, was applied. Accordingly, the protein levels of key ECM components and autophagy were compared across the normal, CQ, GAA, and CQ + GAA treatment groups, highlighting the regulatory effects of GAA on autophagy-mediated degradation pathways (Fig. 6D). Notably, GAA treatment increased the expression of the autophagy-initiated protein Beclin1 while reducing the anti-apoptosis protein Bcl-2, whereas CQ treatment reversed these effects, confirming effective inhibition of autophagic flux. To further elucidate the role of lysosomal degradation in fibronectin processing, Western blot analysis was performed to evaluate the impact of CQ treatment in the presence of TGF-β and GAA. The results demonstrated that CQ blocked autophagic flux and prevented fibronectin degradation. Consistently, Immunofluorescence analysis of fibronectin and lysosomal marker LAMP2 in HCSCs revealed significant colocalization following TGF-β and GAA treatment. Notably, CQ mediated lysosomal dysfunction led to increased accumulation and colocalization of fibronectin with LAMP2, indicating GAA promotes fibronectin degradation through the lysosomal pathway (Fig. 6F). Collectively, GAA regulated the homeostasis of pro-fibrotic cytokines, limiting the inflammatory response that perpetuates fibrosis. These effects collectively contributed to a more regulated wound-healing process with reduced scarring potential.

**Fig. 7:**
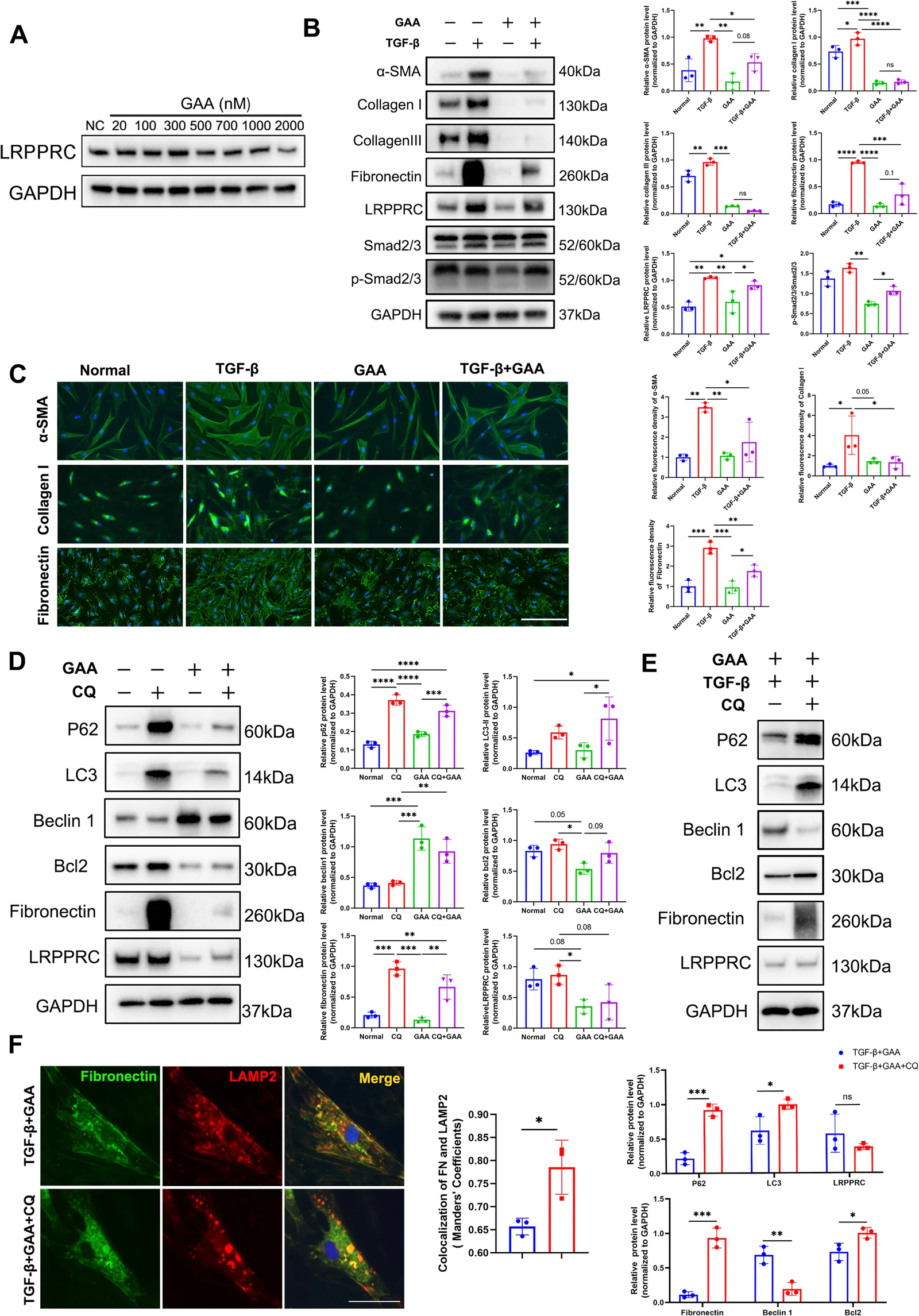
GAA suppresses TGF-β-mediated fibrosis in HCSCs. (**A**) Immunoblotting of indicated LRPPRC in HCSCs. HCSCs were pretreated with GAA at different concentrations for 7 days (*n* = 3). (**B**) Western blot analysis of GAA suppresses TGF-β-mediated fibrosis in HCSCs (*n* = 3). (**C**) Immunostaining of α-SMA, collagen I and fibronectin in HCSCs; scale bars, 100 μm. Relative Fluorescence intensities were quantified using ImageJ software (*n* = 3); scale bar: 100 μm. (**D**) Western blot analysis showed that GAA promoted autophagy in HCSCs (*n* = 3). (**E**) Western blot analysis showed that CQ blocked GAA-mediated autophagy(*n* = 3). (**F**) Immunoblotting of indicated CQ blocked lysosomal degradation. (*n* = 3), scale bar: 50 μm. GAA:2µM; TGF-β:10µM; CQ:10µM. The presented data depict the average and standard deviation from a minimum of three repetitions, with differences assessed using standard one-way ANOVA. *p < 0.05, **p < 0.01, ***p < 0.001

## Discussion

The corneal stroma constitutes 90% of the corneal thickness and is essential for maintaining vision, corneal transparency and refractive function. Consequently, any damage to the corneal stroma due to injury or disease may result in irreversible loss of vision. During corneal wounds, infections and other corneal pathologies, quiescent stromal cells are activated and differentiate into fibroblasts and myofibroblasts. Corneal fibroblasts synthesize and secrete ECM components, including fibronectin, α-SMA, integrins, collagen I and collagen III, thereby facilitating corneal wound healing [20]. However, during the healing process, excessive activation of fibrocytes leads to excessive ECM secretion, which often accumulates proteins with abnormal composition and structure. Over time, these proteins can disrupt the fibrous tissue, leading to persistent scarring and resulting in corneal opacification [21]. The right amount of corneal stromal cell activation and restoration of normal corneal ECM structure are crucial strategies for promoting corneal regeneration and restoring transparency [22].

Corneal fibrosis is predominantly driven by activation of the classical TGF-β signaling pathway. TGF-β and its downstream effectors promote fibroblast differentiation, myofibroblast formation, and resistance to apoptosis, thereby sustaining fibrotic remodeling [1, 23]. Both corneal epithelial and stromal cells express TGF-β isoforms (TGF-β1, TGF-β2, and TGF-β3) and their receptors. TGF-β stimulates corneal epithelial cell migration via integrin β1, mediating the activation of the p38 MAPK pathway, ECM expression and epithelial-mesenchymal transition (EMT) [24, 25]. Consequently, inhibiting TGF-β expression and signaling is a potential approach to combat fibrotic changes [26]. In alkali-burned corneas, VEGF blockade with bevacizumab also inhibited TGF-β expression and improved corneal clarity [27]. Novel anti-TGF-β therapies are emerging as part of routine treatments for wound healing. Similarly, we observed that TGF-β/Smad pathway was suppressed after LRPPRC inhibition, also leading to decreased ECM synthesis. This finding confirms the potential of LRPPRC to improve the prognosis of corneal damage repair.

In addition to profibrotic signaling, emerging evidence indicates that autophagy plays a critical role in maintaining ECM homeostasis and facilitating effective wound healing. Autophagy is vital for maintaining ECM homeostasis and facilitating wound healing. Autophagy is a cellular process in which isolation membranes are formed to enclose cytoplasmic components that necessitate degradation, including damaged organelles, misfolded proteins and aggregated proteins. These structures, known as autophagosomes, subsequently fuse with lysosomes, leading to the degradation of all enclosed substrates within the resulting autolysosomes [28]. It plays a protective role against tumors and neurodegenerative diseases and is also involved in delaying aging, extending lifespan, and combating pathogen infections. The degradative function of autophagy is crucial for normal organismal development and responses to various environmental stresses. Dysregulation or delays in autophagy can negatively impact ECM dynamics and wound-healing processes, potentially resulting in corneal dysfunction. In the present study, we demonstrated that the expression of fibronectin, α-SMA, integrin β1, collagen I and collagen III was significantly up-regulated in corneal tissue during corneal repair after alkali burn, indicating that ECM deposition and corneal fibrosis were increased. Notably, autophagic activity was transiently increased during the early phase of corneal injury but declined during the fibrotic stage of repair, suggesting that insufficient autophagy at later stages may compromise effective degradation of excessive ECM components and thereby promote fibrosis.

**Fig. 8:**
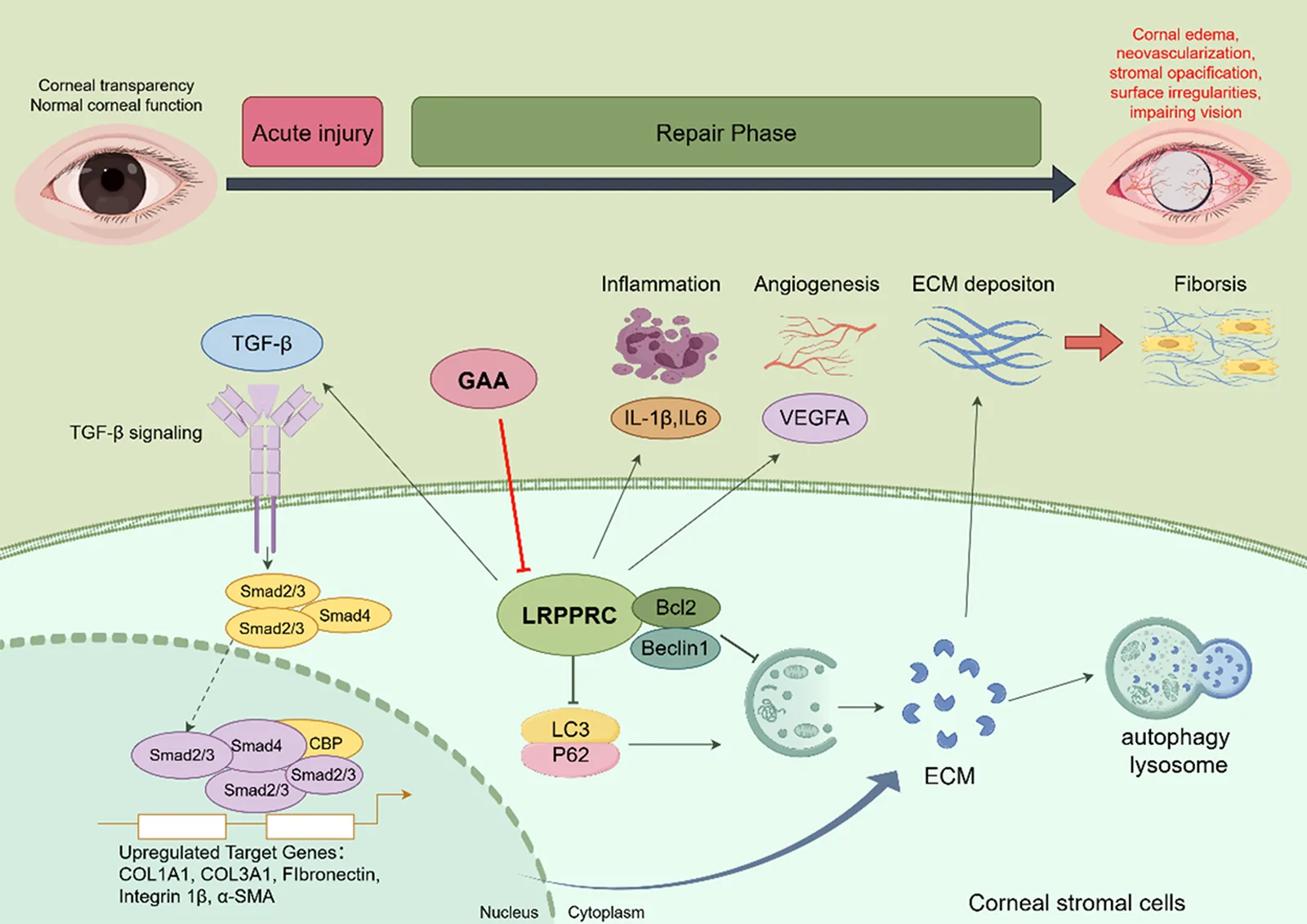
Schematic representation of the mechanism by which inhibition of LRPPRC affects corneal alkali burn repair. Healthy corneas are transparent and have normal refractive function. Following acute injury, the corneal repair process is initiated. LRPPRC promotes activation of the TGF-β/Smad signaling pathway, inhibits autophagy, and stimulates the production of inflammatory factors and VEGFA, thereby facilitating ECM deposition, fibrosis, inflammation, and neovascularization ultimately resulting in corneal edema, opacity, and visual impairment. GAA, a natural drug, alleviates these adverse outcomes by inhibiting LRPPRC and blocking this pathway (By Figdraw)

LRPPRC is a mitochondria-associated protein, and mutations in its gene result in Leigh syndrome French-Canadian type (LSFC), a disorder characterised by neuronal necrosis and defects in cytochrome C oxidase activity [29]. LRPPRC is also involved in embryonic development, mitochondrial function regulation, negative regulation of autophagy and mitosis, and tumorigenesis, acting as an oncogene [30–32]. LRPPRC is closely associated with the development of gastric, prostate and ovarian cancers, and may serve as a prognostic marker for these malignancies [24–26]. Additionally, LRPPRC interacts with the mitochondrial autophagy-related protein Parkin to form a complex, stabilizing Parkin and inhibiting mitochondrial autophagy. LRPPRC forms a stable complex with Bcl-2 and Beclin1, regulating the dissociation of Bcl-2 from Beclin1 and the interaction between Beclin1 and PI3KCIII, thereby modulating the activation of the PI3K/Akt/mTOR signaling pathway [14–16]. In our study, we demonstrate that LRPPRC is a key negative regulator of autophagy. By promoting autophagy, LRPPRC inhibition facilitated ECM degradation and reduced its pathological accumulation. In the corneal alkali burn mouse, knockdown of LRPPRC significantly reduced the expression of pro-inflammatory cytokines IL-1β and IL-6, as well as VEGFA production, thereby attenuating inflammatory responses and neovascularization, both of which contribute to stromal opacity and impaired vision. However, the precise mechanisms by which LRPPRC regulates inflammatory cytokine expression and angiogenic signaling warrant further investigation. Collectively, these effects alleviated corneal stromal fibrosis during the repair phase, reduced corneal opacity, improved corneal transparency with restored tissue architecture, and ultimately enhanced visual function (Fig. 8). Together, these findings establish a mechanistic link between LRPPRC inhibition, suppression of TGF-β/Smad signaling, activation of autophagy, and enhanced ECM clearance during corneal wound healing.

Gossypol acetic acid (GAA) has been identified as a targeted inhibitor of LRPPRC and has demonstrated notable anti-tumor activity [33, 34]. GAA is a naturally occurring polyphenolic compound extracted from mature cotton seeds and has been used for over three decades as a male contraceptive and in gynaecological treatments in several countries, including the United States [35]. Moreover, 5 µM GAA significantly inhibits oxidative stress-induced retinal pigment epithelial cell death [36]. At concentrations of 20 or 50 µM, GAA effectively mitigates ferroptosis induced by Fe-SP without impacting cardiomyocyte viability [37]. In our study, topical application of 20 µM GAA via eye drops significantly improved the repair and prognosis of alkali burn-induced corneal injuries, reducing corneal stromal deposition, opacity and neovascularization. Currently, there is a lack of effective drugs for reducing corneal scarring in clinical practice. Although anti-TGF-β drugs, glucocorticoids, and growth factors, such as losartan, dexamethasone and recombinant human basic fibroblast growth factor(rh-bFGF), have shown some therapeutic benefit, each is associated with limitations or adverse effects. By contrast, GAA offers advantages including low toxicity, natural origin, and ease of preparation process. While further pharmacokinetic and safety evaluations are required, our findings suggest that topical GAA represents a feasible and promising therapeutic strategy for targeting LRPPRC, modulating stromal fibrosis, and promoting scar-free corneal repair.

## Conclusion

In conclusion, suppressing LRPPRC expression in the cornea inhibits the TGF-β/Smad pathway and promotes autophagy, thereby reducing fibroblast activation, enhancing ECM degradation, and reducing corneal inflammation, fibrosis and neovascularization. This study provides a new theoretical basis for the treatment of corneal stromal injuries offering significant clinical application potential.

## Supplementary Information

Below is the link to the electronic supplementary material.


Supplementary Material 1



Supplementary Material 2



Supplementary Material 3



Supplementary Material 4



Supplementary Material 5



Supplementary Material 6



Supplementary Material 7



Supplementary Material 8



Supplementary Material 9



Supplementary Material 10



Supplementary Material 11


## Data Availability

The data that support the findings of this study are available from the corresponding author upon reasonable request.
