# Supplementary material for: Targeting LRPPRC to improve corneal wound healing via augmenting autophagy and suppressing TGF-β/Smad signaling post-alkali burn
Source: J Transl Med. 2026 Jul 24;24:1121. doi: 10.1186/s12967-026-08653-6 (PMC13531831; doi:10.1186/s12967-026-08653-6)
Supplement: Supplementary file 1 [file 12967_2026_8653_MOESM11_ESM.docx]

Supplementary Figure 1(A) Representative Western blot images showing the protein expression levels at different time points after AAV injection in corneal tissues, along with the corresponding quantification(n=3).(B) Statistical analysis of Western blot quantification data, normalized to the internal control.(C) Representative immunofluorescence images of IL-1β and IL-6 expression in corneas from saline and GAA treated groups, together with quantitative analysis of fluorescence intensity. scale bars, 10μm. Fluorescence intensities were quantified using Image J software (n=3). The presented data depict the average and standard deviation from a minimum of three repetitions, with differences assessed using standard one-way ANOVA. *p < 0.05, **p < 0.01, ***p < 0.001.
